# Efficacy of Pericapsular Platelet‐Rich Plasma Injection Combined With Brachial Plexus Block–Assisted Manipulation in Adhesive Capsulitis: A Randomized Controlled Trial

**DOI:** 10.1155/prm/8613079

**Published:** 2026-08-29

**Authors:** Shuifen Yang, Yun Ye, Fengxia Wang, Hui Huang, Hao Wu, Wenke Zhang, Dan Luo, Minglong Zhang, Guoping Song, Wanyun Zhang

**Affiliations:** ^1^ The Department of Rehabilitation Medicine, The Fourth People’s Hospital of Guiyang, Guiyang 550000, Guizhou Province, China; ^2^ Bone Diseases and Center for Healthy Aging, Department of Medicine III & University Clinic Dresden, University Hospital Carl Gustav Carus, Technische Universität Dresden (TU Dresden), Dresden, Germany, uniklinikum-dresden.de; ^3^ Orthopedic Department of Joint and Trauma, The Fourth People’s Hospital of Guiyang, Guiyang 550000, Guizhou Province, China; ^4^ Beijing Jishuitan Hospital Guizhou Hospital, Guiyang 550000, Guizhou Province, China; ^5^ The Department of Pain Management, The Fourth People’s Hospital of Guiyang, Guiyang 550000, Guizhou Province, China; ^6^ Department of Genetics, Qiqihar Medical University, Qiqihar, Heilongjiang Province, China, qmu.edu.cn

**Keywords:** adhesive capsulitis, manipulation under brachial plexus block, platelet-rich plasma, randomized controlled trial, shoulder pain and function

## Abstract

Adhesive capsulitis causes severe shoulder pain and functional limitation, and single treatment modalities often fail to prevent relapse after manipulation under anesthesia. In this randomized controlled trial, we examined whether adding ultrasound‐guided multitarget platelet‐rich plasma injections to manipulation under brachial plexus block could improve pain, function, and health‐related quality of life. We enrolled 68 adults with adhesive capsulitis confirmed by magnetic resonance imaging and randomly assigned them to manipulation plus standardized rehabilitation or to the same regimen plus pericapsular platelet‐rich plasma injections at baseline, two weeks, and one month. Blinded assessors evaluated outcomes before treatment and at one day, 2 weeks, 1 month, and 3 months. The primary endpoint was shoulder pain on the visual analogue scale at end. Secondary endpoints included the Disabilities of the arm, shoulder, and hand questionnaire; the Oxford shoulder score, the 36‐Item Short Form Health Survey; the Constant Murley shoulder score, and serum inflammatory marker levels. The combined strategy led to faster and more pronounced pain relief, with clinically meaningful differences between group immediately after treatment and at the 3‐month follow‐up. In addition, patients who received platelet‐rich plasma showed greater gains in shoulder function and health‐related quality of life, and a higher proportion of them reached predefined thresholds for clinically important improvement. Short‐term changes in inflammatory markers were exploratory and nonspecific and should not be interpreted as evidence of a causal immunomodulatory effect. Taken together, these findings suggest that multitarget platelet‐rich plasma combined with manipulation under brachial plexus block was well tolerated in this trial and was associated with significant short‐term improvement in pain and function for patients with adhesive capsulitis.

## 1. Introduction

Adhesive capsulitis (AC) is a condition characterized by shoulder pain and restricted range of motion (ROM) due to chronic inflammation of periarticular soft tissues [1]. Commonly termed “frozen shoulder,” this condition typically presents with progressive stiffness. AC predominantly affects individuals around 50 years of age, with an estimated prevalence of 3%–5% in the general population. Its clinical hallmarks include pain and limitations in both active and passive shoulder movement. The condition is often self‐limiting, with many patients improving within 1–2 months during the acute phase. However, those with severe pain and significant motion loss may experience a protracted course lasting 1–2 years [2], and some develop long‐term symptoms. Studies indicate that 20%–40% of patients who are managed conservatively continue to report persistent shoulder pain and functional impairment [3]. The pathophysiology involves the release of inflammatory mediators [4] around the joint capsule, promoting periarticular adhesions and secondary tissue injury, frequently affecting the supraspinatus, infraspinatus, and deltoid muscles. Epidemiological data suggest that approximately 15% of patients are left with lasting functional deficits, often accompanied by sleep disturbances, anxiety, or depression, substantially diminishing quality of life. Thus, key therapeutic goals include early pain control, maximal restoration of shoulder function, and improvement in quality of life.

Current management strategies encompass oral nonsteroidal anti‐inflammatory drugs (NSAIDs), structured rehabilitation, intraarticular corticosteroid injection, manipulation under anesthesia (MUA) [5], and, in severe or refractory cases, arthroscopic capsular release. Among these, the ultrasound‐guided brachial plexus block is widely employed to facilitate MUA [6]. However, single‐modality interventions have limitations because post‐MUA inflammatory edema of pericapsular tissues and renewed mediator release can lead to recurrent capsular adhesion, stiffness, motion restriction, and chronic pain. Platelet‐rich plasma (PRP)—an autologous platelet concentrate obtained via centrifugation—contains numerous growth factors that synergistically promote cell proliferation, differentiation, and tissue repair, while exerting anti‐inflammatory, proangiogenic, and reparative effects [7]. Based on this mechanistic rationale, we combined pericapsular PRP injection with MUA under ultrasound‐guided brachial plexus block to enhance therapeutic outcomes and reduce recurrence. Given the limited clinical evidence supporting this combined approach, our objective was to evaluate its effectiveness and safety, thereby providing practical evidence to support this treatment option for AC management [8].

## 2. Materials and Methods

### 2.1. Study Population

This was a single‐centre, assessor‐blinded, parallel‐group randomized controlled trial (RCT). The study was approved by the Ethics Committee of the Fourth People’s Hospital of Guiyang (approval No. 2025053) and conducted in compliance with the Declaration of Helsinki (1964) and its 2013 revision. Trial registration was completed retrospectively with the Chinese Clinical Trial Registry (registration No. ChiCTR2500110516). The full trial protocol is publicly accessible via the registry database, and the anonymized dataset is available from the corresponding author upon reasonable request. From September 2024 to September 2025, we consecutively enrolled patients clinically diagnosed with AC, confirmed by shoulder magnetic resonance imaging (MRI) grading.

**FIGURE 1:**
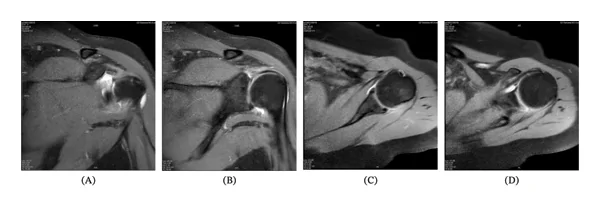
MRI diagnosis of adhesive capsulitis (frozen shoulder) (A) Adhesive capsulitis in the inflammatory phase; LHBT tenosynovitis; mild SASD bursitis. No definite full‐thickness cuff tear. (B) Supraspinatus tendinopathy with a probable small articular‐sided partial‐thickness tear (Ellman Grade I, < 25%–30% thickness). No full‐thickness tear. (C) Supporting adhesive capsulitis; LHBT tenosynovitis; mild subscapularis tendinopathy without definite tear. (D) Rotator interval and coracohumeral ligament involvement—further evidence for adhesive capsulitis; LHBT tenosynovitis. No definite labral tear on this slice.

### 2.2. Inclusion Criteria

1. Clinical diagnosis of AC, characterized by marked restriction of shoulder motion (most pronounced in abduction and external rotation) with shoulder pain; active ROM (AROM) rated as moderate to severe limitation, or near inability to initiate movement (e.g., forward flexion < 10°); passive ROM (PROM) rated as moderate to severe limitation, or nearly absent.
2. Imaging consistent with AC, with Neviaser MRI grading I–II [9] (Figure 1). Patients with clinical stage I–II AC according to the Neviaser staging system, with MRI findings supportive of AC and without full‐thickness rotator cuff tears or other structural lesions requiring surgical treatment.
3. Visual analogue scale (VAS) for pain ≥ 5.
4. Inadequate analgesic response to oral NSAIDs and inability to cooperate with independent rehabilitation training.

### 2.3. Exclusion Criteria

1. History of shoulder trauma with fracture, prior shoulder surgery, or prior intraarticular injection.
2. Calcific tendinopathy of the rotator cuff or other conditions deemed unsuitable for joint manipulation.
3. Serious systemic comorbidities (e.g., tuberculosis, malignancy, hematologic disease, and cardiovascular/cerebrovascular disease) or coagulopathy.
4. Thyroid disease or other conditions potentially affecting treatment or anesthetic safety.

**FIGURE 2:**
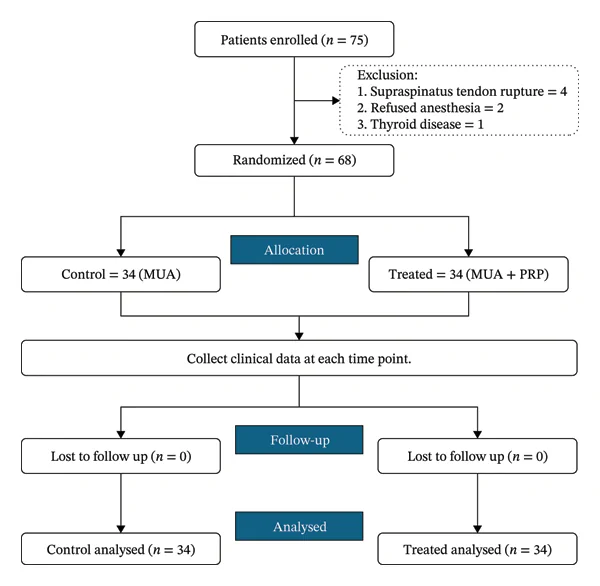
Research approach.

Based on these criteria, 75 patients were initially enrolled and allocated to two groups. The control group received ultrasound‐guided brachial plexus block–assisted joint manipulation and rehabilitation. The experimental group received the same regimen combined with pericapsular PRP injection. In the control group, two patients were excluded due to MRI‐confirmed supraspinatus tendon rupture, and one declined brachial plexus block. In the experimental group, three patients were excluded for supraspinatus tendon rupture on MRI, one refused brachial plexus block, and one had a history of thyroid disease (a relative contraindication to brachial plexus anesthesia). Ultimately, 68 patients were included in the analysis (control *n* = 34; experimental *n* = 34); participant flow is shown in Figure 2.

## 3. Methods

### 3.1. Study Design

Research approach.

### 3.2. Ultrasound‐Guided Brachial Plexus Block

Patients fasted from solid food for 6 h and clear fluids for 2 h before the procedure. After routine skin preparation and sterile draping, the patient was placed supine. A high‐frequency linear transducer was positioned superior to the sternocleidomastoid muscle. Using an in‐plane approach and adjusting the needle trajectory as needed [10], the brachial plexus trunks were visualized clearly and the needle was advanced through the interscalene space between the anterior and middle scalene muscles to achieve brachial plexus block anaesthesia [11].

### 3.3. PRP Preparation and Injection

PRP was prepared using a commercial separating‐gel kit. Forty millilitres of autologous venous blood was collected with 5 mL sodium citrate and processed by two‐step centrifugation (1500 rpm for 10 min, followed by 1800 rpm for 12 min), yielding approximately 5 mL of leukocyte‐poor PRP with an expected platelet concentration 4–6 times that of whole blood. The product was not externally activated and was used within 2 h. Although the standardized protocol minimized procedural variability, platelet and leukocyte counts were not measured for each patient, and interindividual variation in PRP composition cannot be excluded. To maximize the therapeutic effect on the widespread inflammatory cascade typical of AC, we utilized a combined multitarget injection protocol. Rather than an isolated capsular injection, PRP was delivered into both the pericapsular spaces and the adjacent symptomatic peritendinous/bursal structures, customized according to the patient’s MRI findings and physical examination.

**FIGURE 3:**
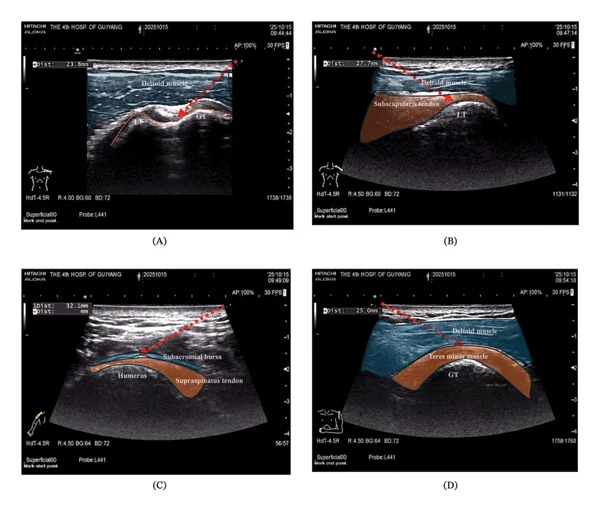
Ultrasound‐guided multitarget shoulder pericapsular injection pathway. (A) Anterior (subcoracoid/rotator‐interval) approach—advance the in‐plane needle to the anterior glenohumeral recess, carefully avoiding the long‐head biceps tendon. (B) Intertubercular groove, short‐axis view—insert the needle in‐plane from lateral to medial and deliver medication into the long‐head biceps tendon sheath (not intratendinous). (C) Posterior approach—in‐plane from superolateral to inferomedial, guide the needle into the posterior glenohumeral recess between the humeral head and posterior labrum. (D) Lateral coronal view—in‐plane from lateral to medial into the subacromial–subdeltoid bursa, creating visible bursal separation on injection. Abbreviations: LT, lesser tubercle; GT, greater tubercle.

All injections were performed under real‐time ultrasound guidance using a 22‐gauge needle and an in‐plane technique. Before each injection, color Doppler was used to scan for blood vessels, and aspiration was performed to confirm the absence of blood. Specifically, the targets included the anterior glenohumeral recess (pericapsular), the posterior glenohumeral recess (pericapsular), the long‐head biceps tendon sheath (peritendinous), and the subacromial–subdeltoid bursa (bursal), corresponding to Figure 3A–D. The injection volume per site ranged from 0.5 to 1.5 mL, tailored to tissue compliance and pathology severity. The total injection volume per session was 4–6 mL. All participants were followed up at baseline, after treatment (Post), 2 weeks (W2), 1 month (M1), and 3 months posttreatment (M3) (Figure 3).

Throughout the follow‐up period, nine adverse events were reported: three in the PRP group and six in the control group. No serious adverse events occurred. The most frequent events were posttreatment pain (5/9, 55.6%) and transient swelling (4/9, 44.4%). All events were mild and self‐limiting and resolved within 48 h without additional intervention.

### 3.4. Manual Rehabilitation (MUA)

**FIGURE 4:**
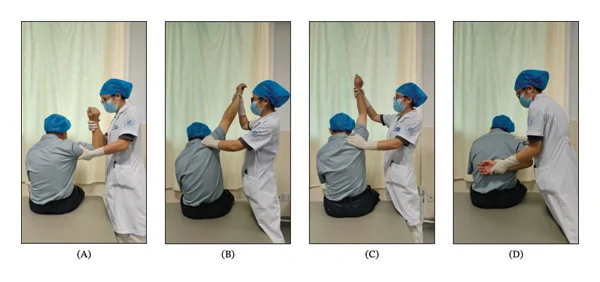
Manipulation under brachial plexus block (A) External rotation mobilization: The clinician stabilized the scapula with one hand and held the patient’s elbow with the other, slowly externally rotating the humerus to the end‐range barrier and holding for 10 s. (B) Abduction mobilization: With the distal clavicle stabilized and the distal humerus supported, the shoulder was abducted to the point of maximal resistance, followed by small‐amplitude oscillatory mobilization at end‐range. (C) Forward flexion mobilization: One hand applied light pressure over the superior medial scapula while the other supported the elbow to deliver longitudinal traction along the humeral axis, holding for 10 s; this maneuver was repeated 3–5 times. (D) Internal rotation mobilization: With the patient prone and the upper limb extended and internally rotated, the clinician stabilized the scapula and moved the forearm medially toward the spine, applying sustained end‐range pressure to increase internal rotation until the forearm reached the level of the T10 spinous process or higher.

Following ultrasound‐guided brachial plexus block, the affected shoulder was stabilized, and structured manual therapy was performed. Interventions prioritized the direction of least resistance, progressed gradually, and avoided excessive force to prevent tissue injury. The protocol included the following (Figure 4).

In the experimental group, pericapsular PRP injection was added to the same MUA protocol performed under brachial plexus anesthesia to enhance anti‐inflammatory and tissue‐repair effects. Both groups were followed at pre‐specified time points through outpatient visits or telephone follow‐up.

### 3.5. Outcomes

Patients in both groups were assessed at preoperative baseline (Pre), postoperative day 1 (Post), postoperative week 2 (W2), postoperative month 1 (M1), and postoperative month 3 (M3). The following outcomes were collected:

1. VAS for pain, scored 0–10 to reflect pain severity.
2. Constant–Murley Score, a composite measure of shoulder function (0–100), with higher scores indicating better function.
3. Oxford shoulder score (OSS), consisting of 12 items each scored from 1 to 5 and yielding a total score ranging from 12 to 60, with lower scores indicating fewer symptoms and better shoulder function.
4. Disabilities of the arm, shoulder, and hand (DASH), evaluating upper‐limb (shoulder, elbow, wrist, and hand) disability and symptoms and their impact on daily life.
5. Short Form‐36 (SF‐36), assessing eight domains of health‐related quality of life, with higher scores positively correlated with better quality of life.
6. Serum inflammatory biomarkers—tumor necrosis factor‐*α* (TNF‐*α*), interleukin‐6 (IL‐6), and interleukin‐10 (IL‐10)—measured at Pre and W2 as indices of sterile inflammatory activity and its improvement.

### 3.6. Randomization and Blinding Procedures

The randomization sequence was generated by an independent researcher not involved in clinical care, using a computer‐generated random‐number table. Participants were randomized in a 1:1 ratio using variable block sizes of 4 and 6, with stratification based on baseline VAS scores. To ensure robust allocation concealment, we employed sequentially numbered, opaque, sealed envelopes (SNOSE). These envelopes were opened by the treating physician only after the participant had fulfilled all inclusion criteria and completed the baseline assessments.

Although the physical nature of pericapsular injections and manual rehabilitation precluded blinding for patients and treating physicians, we maintained strict blinding for all assessors and analysts. An independent rehabilitation evaluator, unaware of group assignments, conducted all follow‐up clinical evaluations, ROM measurements, and questionnaire administrations (VAS, DASH, OSS, SF‐36, and Constant–Murley). Furthermore, blinded laboratory technicians and a statistician performed the blood sample analyses and statistical processing, respectively.

### 3.7. Sample Size Calculation

Assuming 30 completers per arm, the primary analysis was planned to test the between‐group difference at the final follow‐up (M3) with a two‐sided *α* = 0.05. Under plausible assumptions—a final‐timepoint SD of ∼2.0 and a baseline‐to‐final correlation *ρ* ≈ 0.6—30 per group provided ∼80% power to detect *a* ≥ 1.15‐point difference. To buffer attrition, we aimed to enroll at least 34 participants per group. Allocation was 1:1 using variable block sizes (4 and 6), with stratification by baseline VAS.

### 3.8. Statistical Analysis

All analyses and figures were generated using R (R Foundation for Statistical Computing, Austria; 2024.09.1A + 394) and Python (Python Software Foundation, USA; version 3.11.8). Baseline continuous variables conforming to normality were summarized as mean ± standard deviation (SD); between‐group comparisons used the independent‐samples *t* test (for equal variances) or Welch’s *t* test (for unequal variances). For repeated measures while accounting for within‐subject correlation, we fit generalized estimating equations (GEE) models, treating time as a categorical factor with five levels (Pre, Post, W2, M1, and M3). All models were adjusted for potential confounders. The analysis was performed on an intention‐to‐treat (ITT) basis. Although follow‐up was complete for all 68 randomized participants, any potential missing longitudinal data were planned to be handled via the GEE framework using the missing‐at‐random (MAR) assumption.

Model selection was guided by the quasi‐likelihood under the independence model criterion (QIC), with the smallest QIC indicating the preferred working correlation structure. After model fitting, residual normality was evaluated with the Shapiro–Wilk test; a *p* value > 0.05 was interpreted as no significant deviation from normality. Across outcomes, the unstructured working correlation consistently yielded the lowest QIC (e.g., unstructured QIC = 344.65) and was therefore adopted for the final analyses. Model outputs included estimated regression coefficients (*β*), 95% confidence intervals (CI), and *p* values. We also derived covariate‐adjusted means at each time point (least‐squares means) to visualize intervention effects. Pairwise comparisons across time points were multiplicity‐adjusted using Holm procedures. To quantify effect magnitude from baseline to the 3‐month follow‐up, we calculated Cohen’s *d* using an estimator appropriate for paired designs. Clinical significance was further examined by the proportion of participants achieving the pre‐specified responder threshold (used here as a surrogate for the minimum clinically important difference, MCID), defined a priori as ≥ 30% improvement from baseline in the respective outcome. Absolute anchor‐based thresholds were also considered as a sensitivity check (VAS ≥ 2); a percentage‐based MCID was pre‐specified because it scales across differing baseline severities and across the multiple outcome instruments used.

Finally, we compared candidate working correlation structures—unstructured, exchangeable (compound symmetry), independence, and AR (1)—via QIC and confirmed that the unstructured form provided the best fit for all outcomes, consistent with the above (lowest QIC). Residuals for the primary endpoint were approximately normal; minor right skew in some secondary outcomes did not materially affect the robustness of the estimates. Results were robust to this choice: point estimates and inferences were stable across the alternative working correlation structures, in line with the known robustness of GEE estimates to correlation misspecification when combined with standard errors.

## 4. Results

### 4.1. Baseline Characteristics

A total of 68 patients with AC were included. At baseline, the control and experimental groups were comparable in age, sex distribution, mean age, and pain grading; no statistically significant between‐group differences were observed (Table 1).

**TABLE 1:** Demographic and clinical characteristics of patients in two groups.

Characteristic	Category	Control (*n* = 34)		Treated (*n* = 34)		Value (*t*/*χ* ^2^)	*p* value
		Mean ± SD/*n* (%)	95% CI	Mean ± SD/*n* (%)	95% CI		
*Demographics*							
Age		60.03 ± 6.15	[57.88, 62.18]	59.68 ± 5.82	[57.65, 61.71]	−0.24	0.809

Gender	Male	22 (64.7%)		15 (44.1%)		2.90	0.09
	Female	12 (35.3%)	[21.5%, 52.1%]	19 (55.9%)	[39.5%, 71.1%]	2.13	0.144

Disease Duration (Days)		33.56 ± 18.11	[27.24, 39.88]	29.44 ± 18.72	[22.91, 35.97]	0.92	0.360

Location	Left	15 (44.1%)		9 (26.5%)			
	Right	19 (55.9%)	[39.5%, 71.1%]	25 (73.5%)	[56.9%, 85.4%]	1.61	0.205

Diabetes	No	30 (88.24%)		32 (94.12%)		0.18	0.67
	Yes	4 (11.76%)		2 (5.88%)			

Hypertension	No	32 (94.12%)		32 (94.12%)		0.00	1
	Yes	2 (5.88%)		2 (5.88%)			

Cervical Spondylosis	No	34 (100.00%)		33 (97.06%)		0.00	1
	Yes	0 (0.00%)		1 (2.94%)			

*Outcomes*							
VAS		7.12 ± 1.27	[6.67, 7.56]	7.47 ± 1.74	[6.86, 8.08]	0.95	0.345

DASH		75.29 ± 8.88	[72.20, 78.39]	73.88 ± 12.30	[69.59, 78.17]	0.54	0.589

OSS		49.32 ± 3.85	[47.98, 50.67]	50.32 ± 2.70	[49.38, 51.27]	1.24	0.220

SF36		47.65 ± 9.33	[44.39, 50.90]	47.47 ± 6.51	[45.20, 49.74]	0.09	0.928

Constant–Murley		44.60 ± 2.62	[43.69, 45.52]	44.26 ± 2.16	[43.50, 45.01]	0.60	0.554
	Pain Score	5.66 ± 0.83	[5.37, 5.95]	5.42 ± 0.76	[5.15, 5.68]	1.25	0.216
	ADL	9.03 ± 1.27	[8.59, 9.47]	8.89 ± 1.16	[8.49, 9.30]	0.47	0.638
	ROM	19.74 ± 1.23	[19.31, 20.17]	19.88 ± 1.15	[19.48, 20.28]	0.49	0.629
	Muscle Strength (points)	10.17 ± 1.16	[9.77, 10.58]	10.07 ± 1.18	[9.65, 10.48]	0.38	0.708

*Inflammatory Markers*							
IL‐10		5.66 ± 1.61	[5.10, 6.22]	5.84 ± 1.05	[5.48, 6.21]	0.56	0.576

IL‐6		11.89 ± 3.60	[10.63, 13.14]	12.11 ± 3.10	[11.03, 13.20]	0.28	0.782

TNF‐a		35.47 ± 4.40	[33.94, 37.01]	35.33 ± 3.98	[33.94, 36.72]	−0.14	0.890

*Note:* Continuous cells show mean ± SD and 95% CI; categorical cells show ‘*n* (%’). Value displays the test statistic (*t* for Welch *t*‐test; χ^2^ for chi‐square). Disease duration is expressed in Days, and the Constant–Murley subdomain scores (pain score, ADL, ROM, and muscle strength) in points. IL‐6, interleukin‐6; IL‐10, interleukin‐10.

Abbreviations: ADL, activities of daily living; ROM, range of motion; TNF‐*α*, tumor necrosis factor‐*α.*

### 4.2. Comparison of Primary and Secondary Outcomes Across Time Points

GEE analyses (Table 2) showed that baseline VAS was comparable between groups (*p* = 0.383). The group × time interaction was significant immediately after treatment (Post; *β* = 1.707, *p* < 0.001) and at 3 months (M3; *β* = 1.341, *p* = 0.003). However, the interaction effects at 2 weeks (*p* = 0.882) and 1 month (*p* = 0.080) were not significant, suggesting comparable rates of improvement between groups during the intermediate follow‐up phase. In addition, DASH scores were independently associated with pain improvement (*β* = −0.025, *p* = 0.022). To further evaluate group‐specific effects over time, we conducted multiplicity‐adjusted pairwise comparisons at each time point, relying strictly on Holm‐corrected *p* values for all postbaseline clinical interpretations (Table 3).

**TABLE 2:** Results of the GEE model for VAS pain scores, including the effects of group, time, group × time interaction, and covariates.

VAS	*β*	SE	95% CI	*Z* value	*p* value
Interactions	10.383	1.229	7.974∼12.792	8.449	< 0.001
Group: Con (vs tre)	−0.309	0.355	−1.005∼0.386	−0.872	0.383
Time: Post vs pre	−4.612	0.557	−5.703∼−3.520	−8.28	< 0.001
Time: W2 vs pre	−3.71	0.688	−5.058∼−2.362	−5.396	< 0.001
Time: M1 vs pre	−5.103	0.792	−6.655∼−3.552	−6.447	< 0.001
Time: M3 vs pre	−6.115	0.853	−7.786∼−4.444	−7.173	< 0.001
Group × time: Con × post	1.707	0.421	0.881∼2.532	4.052	< 0.001
Group × Time: Con × *W*2	0.068	0.454	−0.823∼0.958	0.149	0.882
Group × time: Con × *M*1	0.776	0.443	−0.093∼1.644	1.75	0.08
Group × Time: Con × *M*3	1.341	0.448	0.463∼2.219	2.993	0.003
DASH	−0.025	0.011	−0.047∼−0.004	−2.285	0.022
OSS	0.001	0.021	−0.040∼0.043	0.055	0.956
SF‐36	−0.01	0.008	−0.025∼0.006	−1.202	0.229
Constant–Murley	−0.015	0.012	−0.039∼0.009	−1.239	0.215

**TABLE 3:** Comparison of between‐group differences across follow‐up.

Variable	Time	Adjusted mean difference (Con−Tre)	95% CI	SE	Wald *z*	*p*	*p* (Holm)
*Primary outcome*							
VAS	Post	1.500	1.187 to 1.813	0.160	9.399	< 0.001	< 0.001
	W2	−0.206	−0.620 to 0.208	0.211	−0.975	0.330	0.330
	M1	0.471	0.048 to 0.893	0.216	2.182	0.029	0.058
	M3	1.147	0.821 to 1.473	0.166	6.904	< 0.001	< 0.001

*Secondary outcomes*							
DASH	Post	−1.500	−3.531 to 0.531	1.036	−1.447	0.148	0.148
	W2	2.882	0.951 to 4.814	0.985	2.925	0.003	0.007
	M1	5.324	3.634 to 7.013	0.862	6.176	< 0.001	< 0.001
	M3	3.382	1.746 to 5.019	0.835	4.051	< 0.001	< 0.001

OSS	Post	5.500	4.182 to 6.818	0.673	8.177	< 0.001	< 0.001
	W2	3.529	2.260 to 4.799	0.648	5.448	< 0.001	< 0.001
	M1	7.441	6.268 to 8.614	0.598	12.436	< 0.001	< 0.001
	M3	5.647	4.565 to 6.729	0.552	10.229	< 0.001	< 0.001

SF36	Post	−3.441	−6.697 to −0.186	1.661	−2.072	0.038	0.038
	W2	−4.794	−8.195 to −1.393	1.735	−2.763	0.006	0.011
	M1	−4.706	−6.994 to −2.418	1.167	−4.031	< 0.001	< 0.001
	M3	−8.412	−10.845 to −5.978	1.242	−6.775	< 0.001	< 0.001

Constant Murley	Post	−1.721	−2.971 to −0.471	0.638	−2.698	0.007	0.007
	W2	−3.915	−5.391 to −2.440	0.753	−5.200	< 0.001	< 0.001
	M1	−5.664	−7.299 to −4.029	0.834	−6.791	< 0.001	< 0.001
	M3	−7.595	−9.425 to −5.765	0.934	−8.135	< 0.001	< 0.001

*Note:* Con, control group; Tre, treatment group; SF‐36, 36‐Item Short Form Health Survey; Constant–Murley, Constant–Murley shoulder score.

^1^The between‐group effect is defined as the mean difference Con − Tre (the adjusted control‐group mean minus the adjusted treatment‐group mean at each time point; per Note 4, a positive value on lower‐is‐better outcomes and a negative value on higher‐is‐better outcomes favors the experimental group). Values are reported as mean difference (95% CI); *p* values are two‐sided, with *p* < 0.001 displayed as “< 0.001”. Multiplicity control: within each outcome, tests across the postbaseline time points (Post, W2, M1, and M3) are adjusted using the Holm procedure.

^2^The table reports Holm‐adjusted *p* values as *p* (Holm), with *p* < 0.001 displayed as “< 0.001”. Robust inference: sandwich (heteroscedasticity‐robust) standard errors and Wald *z* statistics are reported, with the sign of the statistic aligned to the reported effect direction Effect (Con − Tre).

^3^All estimates in Table 3 are estimated marginal‐mean contrasts (Con − Tre) obtained from a single GEE fit for each outcome (Group × Time, unstructured working correlation) with robust (sandwich) standard errors; the 95% CI, Wald *z*, and raw *p* in each row derive from that same contrast. The Holm procedure is applied across the four postbaseline time points (Post, W2, M1, and M3) within each outcome, so the Holm‐adjusted *p* is never smaller than the corresponding raw *p*.

^4^Direction of benefit: for outcomes where lower scores indicate improvement (VAS, Pain_Score, DASH, and OSS), Effect (Con − Tre) > 0 favors the experimental group. For outcomes where higher scores indicate improvement (SF‐36, Constant–Murley, ADL, ROM, and MuscleStrength), Effect (Con − Tre) < 0 favors the experimental group.

^5^Abbreviations: ADL, activities of daily living; CI, confidence interval; DASH, disabilities of the arm, shoulder, and hand; GEE, generalized estimating equations; OSS, Oxford shoulder score; ROM, range of motion; SE, standard error; VAS, visual analogue scale.

**FIGURE 5:**
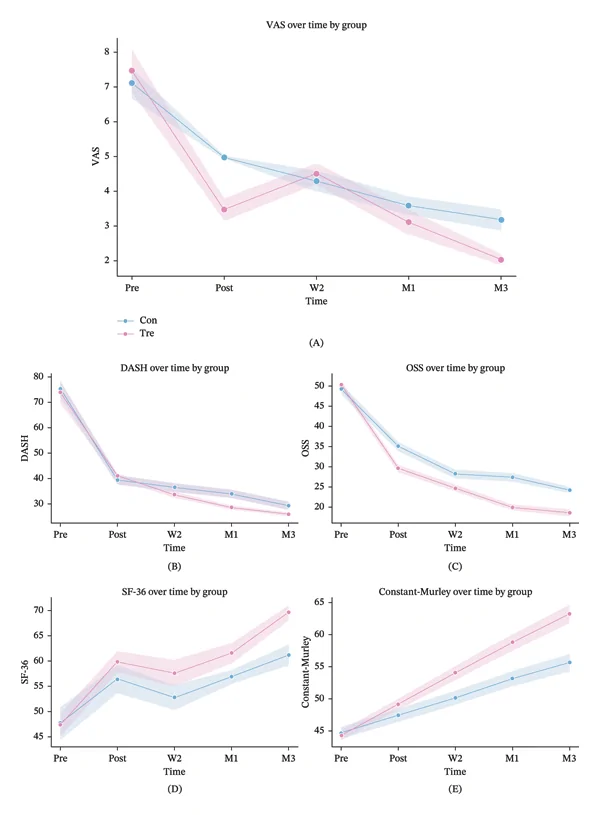
Primary outcome (A) and secondary outcomes (B–E) between control group and treated group.

GEE analyses indicated a time‐varying response to the intervention (Table 3). For the primary outcome (VAS), the treatment group showed an immediate and clinically meaningful reduction in pain after the procedure (adjusted mean difference [Con − Tre] = 1.500; 95% CI 1.187 to 1.813; *p* < 0.001). The between‐group difference was not significant at week 2 (0.206; 95% CI −0.620 to 0.208; *p* = 0.330), and at month 1 it reached nominal significance but did not survive multiplicity adjustment (0.471; 95% CI 0.048 to 0.893; raw *p* = 0.029, Holm‐adjusted *p* = 0.058); the advantage re‐emerged by month 3 with sustained pain relief (1.147; 95% CI 0.821 to 1.473; *p* < 0.001; Figure 5A).

For secondary outcomes, the between‐group difference in DASH was not significant immediately posttreatment (*p* = 0.148); however, beginning at week 2, the treatment group outperformed the control group with statistically significant improvements at all subsequent assessments (all *p* ≤ 0.009; Figure 5B), and the magnitude of benefit increased over time. The OSS consistently favored the treatment group at every follow‐up (all *p* < 0.001), with the largest between‐group difference observed at month 1 (absolute difference = 7.441; Figure 5C). For SF‐36, a significant advantage for the treatment group was evident immediately after treatment (*p* = 0.038) and was maintained—and progressively widened—at week 2, month 1, and month 3 (all *p* ≤ 0.045), peaking at month 3 (Effect = −8.412; Figure 5D). Similarly, Constant–Murley scores were significantly better in the treatment group at all time points (all *p* ≤ 0.01), with the greatest functional gain observed at month 3 (Effect = −7.595; Figure 5E).

After Holm adjustment for multiple comparisons, most time‐point differences remained statistically significant (*p* (Holm) < 0.05; Table 3), with the exceptions of VAS at week 2 and month 1 and DASH immediately posttreatment, which no longer met the significance threshold (Table 3).

### 4.3. Two‐Week Comparison of Inflammatory Markers

**FIGURE 6:**
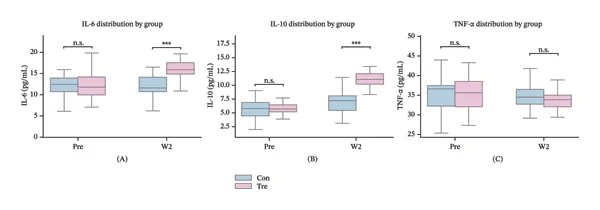
Group’s distributions of inflammatory cytokines at baseline and 2 weeks: IL‐6, IL‐10, and TNF‐*α*.

At W2, serum IL‐10 and IL‐6 concentrations were higher in the treatment group than in the control group (Table 4; Figure 6A, B), whereas TNF‐*α* concentrations did not differ materially between groups (Table 4; Figure 6C). Because these analyses were exploratory and included only two serum sampling time points, the biological significance of these changes remains uncertain. In particular, the increase in IL‐6 may reflect procedure‐related inflammation, platelet‐associated responses, or other nonspecific factors.

**TABLE 4:** Comparison of inflammatory biomarkers between the two patient groups.

Inflammatory markers	Control		Treated	
	Pre	W2	Pre	W2
IL‐6	11.89 ± 3.60	12.04 ± 2.29^∗^	12.11 ± 3.10	15.97 ± 2.23^∗#^
IL‐10	5.66 ± 1.61	6.89 ± 2.41^∗#^	5.84 ± 1.05	11.04 ± 2.11^∗#^
TNF‐a	35.47 ± 4.40	34.40 ± 3.26	35.33 ± 3.98	34.05 ± 3.42

*Note:* 1. Values are reported as mean ± SD with two decimal places. 2. Between‐group comparisons at the same time point (Con vs Tre) use the independent‐samples *t* test; within‐group comparisons (W2 vs Pre) use the paired *t* test. 3. Statistical significance at *p* < 0.05 is denoted by “∗” for between‐group differences and “#” for within‐group changes.

## 5. Endpoint Improvements Relative to Baseline

**FIGURE 7:**
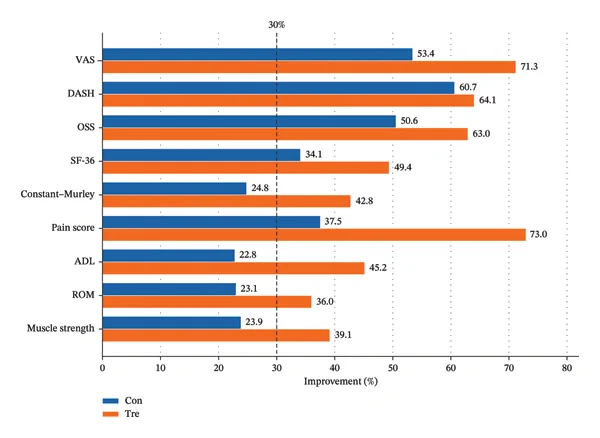
Mean percentage improvement from baseline to 3 months by group.

Across all endpoints, the Tre group outperformed the Con group (Table 5; Figure 7). Mean improvement was higher with Tre—VAS 71.3% vs 53.4% and Pain Score 73.0% vs 37.5%. Most participants achieved the prespecified ≥ 30% responder threshold for VAS, DASH, and OSS (VAS: Con 33/34, Tre 34/34), whereas MCID attainment was lower in Con for SF‐36, ADL, ROM, and muscle strength. Overall, Tre showed consistently higher MCID rates, indicating greater clinical benefit.

**TABLE 5:** Improvements relative to baseline at third month.

Outcomes	Control		Treated		Effect size (Cohen’s *d*)
	Mean improvement (%)	Control responders (≥ 30%)	Mean improvement (%)	Treated responders (≥ 30%)	
VAS	53.44	33	71.27	34	−0.86
DASH	60.71	34	64.08	34	−0.19
OSS	50.58	34	62.99	34	−1.72
SF36	34.13	15	49.44	26	0.87
Constant Murley	24.85	9	42.82	34	2.79
Pain Score	37.5	23	72.96	34	1.84
ADL	22.78	7	45.18	33	2.27
ROM	23.07	9	36.04	23	1.16
Muscle strength	23.86	8	39.13	27	1.5

*Note:* For each group—control (Con) and treatment (Tre)—the mean percentage improvement from baseline to 3 months (M3) was computed as follows: for outcomes where lower scores indicate improvement (VAS, DASH, and OSS), improvement = (Pre − M3)/Pre × 100%; for outcomes where higher scores indicate improvement (SF‐36, Pain Score, Constant–Murley and its subdomains, ADL, ROM, Muscle Strength), improvement = (M3 − Pre)/Pre × 100%. *A* ≥ 30% improvement from baseline was pre‐specified as the responder threshold and is used here as a surrogate for the minimum clinically important difference (MCID). Effect sizes for baseline‐to‐M3 change were estimated with paired‐sample Cohen’s *d*; signs reflect the direction of change (negative denotes a decrease from baseline where lower is better), and absolute values are reported for ease of interpretation.

Effect sizes (Cohen’s *d* on change from baseline to 3 months; positive favors Tre on higher‐is‐better scales, negative favors Tre on lower‐is‐better scales) were: VAS −0.86, DASH −0.19, OSS −1.72, SF‐36 + 0.87, Constant–Murley +2.79, pain score +1.84, ADL +2.27, ROM +1.16, and muscle strength +1.50. Most outcomes showed large to very large effects (|*d*| ≥ 0.8). The very large Constant–Murley effect (*d* = 2.79) reflects its composite construction—aggregating pain, activities of daily living (ADL), ROM, and strength—so concurrent gains across subdomains compound; it should be read as a composite signal rather than a single‐construct effect.

Relative risks (Tre vs Con) for achieving MCID further quantified this advantage: VAS 1.03 (95% CI 0.97–1.09); DASH 1.00 (both groups 100%); OSS 1.00 (both groups 100%); SF‐36 1.73 (1.14–2.64); Constant–Murley 3.78 (2.16–6.62); pain score 1.48 (1.17–1.87); ADL 4.71 (2.43–9.15); ROM 2.56 (1.39–4.69); muscle strength 3.37 (1.80–6.33). For SF‐36, Constant–Murley, ADL, ROM, and muscle strength, RRs were well above 1, indicating Tre participants were roughly 1.7–4.7 times more likely to achieve clinically meaningful improvement. In a prespecified sensitivity analysis using an absolute anchor‐based threshold (a VAS reduction of ≥ 2 points from baseline to 3 months), responder rates were 33/34 (97.1%) in the control group and 34/34 (100%) in the treatment group (RR 1.03, 95% CI 0.97–1.09; Fisher exact *p* = 1.00); the near‐ceiling response in both arms indicates limited discrimination at this threshold and The near‐ceiling response rates in both groups indicated limited between‐group discrimination at this absolute threshold.

## 6. Discussion

AC stems from chronic injury to periarticular shoulder soft tissues [1]. Key pathological changes include glenohumeral capsular fibrosis with adhesive contracture [12], tenosynovitis of the long head of the biceps, and subacromial–subdeltoid bursitis [13]. Varying degrees of rotator cuff tendon involvement are common, exacerbating pain and loss of ROM. As a diagnostic adjunct, MRI offers advantages over computed tomography and ultrasound in acute and subacute phases, particularly for detecting capsular thickening and rotator cuff tears [14]. The MRI‐based Neviaser grading system [9], which assesses the severity of capsular involvement, can also aid in formulating individualized treatment strategies.

From a clinical perspective, early AC is associated with low‐grade sterile inflammation of the shoulder capsule and surrounding periarticular tissues. Previous studies have suggested that inflammatory mediators, including IL‐6 and TNF‐*α* [15], and profibrotic pathways involving TGF‐*β*, may participate in extracellular‐matrix remodeling [16]. These processes may contribute to capsular thickening, reduced tissue compliance, and progressive pericapsular fibrosis. Clinically, such pathological changes manifest as persistent shoulder pain, stiffness, and functional limitation, which represent the major therapeutic targets in AC.

Clinical AC management is diverse and lacks a universally accepted standard [17]. Common approaches include oral NSAIDs in the acute phase, corticosteroid injections, joint mobilization or manipulation, capsular release, and structured rehabilitation. For persistent disease, prolonged symptoms, or MRI [18] findings suggestive of Neviaser Grade III, arthroscopic capsular release (sometimes with capsular repair) may be considered, highlighting the importance of early intervention in these cases. RCT evidence indicates that intraarticular corticosteroid injections can reduce shoulder pain [2], but do not reliably restore ROM, whereas joint mobilization may improve motion, yet fail to provide durable pain relief [19]. Some patients experience repeated capsular inflammation and prolonged stiffness, missing the optimal treatment window and ultimately requiring surgery [20]—further increasing patient and societal burden.

To improve outcomes, intraarticular PRP injection has emerged as a promising AC treatment option [21]. In this study, patients in both the control and treatment groups at the acute and subacute stages underwent MUA [11] with ultrasound‐guided brachial plexus block [22, 23]. The treatment group additionally received ultrasound‐guided pericapsular PRP injections. Ultrasound guidance enabled targeted, image‐guided pericapsular delivery, allowing PRP to reach inflamed lesions more precisely, which was reflected in favorable clinical responses [18, 22].

PRP contains platelet‐derived growth factor, transforming growth factor‐*β*, and other growth factors that may be involved in inflammation regulation and tissue remodeling [24]. These properties provide a biological rationale for its use in AC, although the underlying mechanism remains exploratory in the present study. Because this trial did not include a corticosteroid comparison group, no conclusion can be drawn regarding the relative efficacy of PRP versus corticosteroids. Although literature indicates PRP’s potential for superior tissue remodeling compared to corticosteroids, our lack of a direct comparative design necessitates further research to delineate its relative efficacy [25].

Consistent with our clinical findings, although VAS differences at week 2 (*p* = 0.330) and, after multiplicity adjustment, month 1 (Holm‐adjusted *p* = 0.058) were not significant, the PRP group showed superior pain relief immediately after treatment (*p* < 0.001) and at 3 months (*p* < 0.001). As AC management must simultaneously address pain and prevent capsular adhesion progression, we also evaluated upper‐limb function and general daily activities over 3 months. The treatment group demonstrated greater improvements in several functional outcomes. However, the present clinical trial did not directly assess capsular healing, tissue perfusion, tendon repair, or local inflammatory resolution; therefore, these clinical findings should not be interpreted as evidence of a specific biological mechanism. Moreover, Constant–Murley subdomain analysis (pain, ROM, ADL, and muscle strength) consistently favored the treatment group, suggesting PRP may be valuable for chronic or refractory shoulder pathology [26]. Notably, the effect size for the Constant–Murley score (*d* = 2.79) at the 3‐month follow‐up was exceptionally large. This can be explained by the nature of the Constant–Murley score as a composite metric that integrates pain, ADL, ROM, and muscle strength. Because the intervention produced substantial and concurrent improvements across all these subdomains, their combined clinical benefit significantly amplified the overall mean difference. Thus, the observed large effect size may be attributable to synergistic improvements across multiple domains, combined with relatively consistent treatment responses among participants. The nonmonotonic VAS trajectory should be interpreted cautiously. The immediate between‐group difference, its attenuation at W2 and M1, and its re‐emergence at M3 may reflect a combination of procedural effects, natural symptom fluctuation, regression toward the mean, repeated treatment timing, and sampling variability. Although a delayed treatment‐associated effect is possible, the present study was not designed to establish a reparative mechanism.

At the 2‐week mark, a notable fluctuation in serum inflammatory markers was observed, with the treatment group exhibiting significantly higher levels of IL‐6 and IL‐10 compared to controls. While IL‐10 is a recognized anti‐inflammatory mediator, the elevation of IL‐6 warrants a nuanced interpretation. Despite its proinflammatory role in joint pathology, IL‐6 exerts pleiotropic effects that may support metabolic and regenerative processes. This rise in the PRP group likely reflects a transient biological response to the injection, where platelet degranulation triggers an initial inflammatory cascade before transitioning to the reparative phase [24]. These cytokine findings are exploratory and nonspecific: the IL‐6 elevation may equally reflect procedure‐related inflammation rather than a beneficial immunomodulatory effect, and no causal inference should be drawn.

Historically, evaluating AC via a single dimension—pain relief or functional restoration—has been challenging, as patient symptoms often overlap during the disease course [27, 28]. If substantial pain, motion loss, and disability persist post‐MUA, escalation to open or arthroscopic capsular release is often considered; prior studies suggest benefit [29]. Furthermore, complementing traditional endpoints, we compared groups using the MCID [30] framework for baseline‐to‐endpoint improvement in specific functional and pain measures. Notably, upper‐limb functional gains were more pronounced in the treatment group, underscoring the importance of addressing pain and function concurrently rather than in isolation.

In summary, ultrasound‐guided brachial plexus block–assisted MUA combined with pericapsular PRP injection reduced pain in AC and improved shoulder mobility and health‐related quality of life. However, study limitations include a modest sample size and relatively short follow‐up compared to the typical AC timeline. Longitudinal studies are essential to ascertain the sustained impact and clinical significance of these initial findings. In addition, the serum cytokines measured (IL‐6, IL‐10, and TNF‐*α*) are not acute‐phase reactants and lack established sensitivity and specificity for AC, a condition characterized by low‐grade rather than florid inflammation; the cytokine findings should therefore be regarded as exploratory and hypothesis‐generating. Multicentre studies with larger cohorts and longer observation periods are warranted to confirm these findings and strengthen the clinical decision‐making evidence base.

## 7. Perspective

This work suggests that combining mechanical capsular release with pericapsular PRP injection may be a useful adjunct in the management of AC. Manipulation under brachial plexus block helps restore movement quickly, and the addition of targeted PRP injections was associated with greater short‐term improvement in pain and daily function; the present study was not designed to determine the underlying biological mechanism. Because ultrasound guidance and regional anesthesia are already available in many hospitals, this protocol has practical potential if larger, longer multicentre studies confirm its long‐term benefits, recurrence profile, and the patient groups most likely to benefit.

## Funding

This study was supported by the Guizhou Provincial Health Commission Science and Technology Fund, No. gzwkj2024‐031.

## Conflicts of Interest

The authors declare no conflicts of interest.

## Data Availability

The data that support the findings of this study are available on request from the corresponding authors. The data are not publicly available due to privacy or ethical restrictions.
